# Dr. Keating

**Published:** 1861-05

**Authors:** 


DR. KEATING.—At a meeting of the Trustees of the Jefferson Medical College, on the 8th of April, Dr. William V. Keating, of this city, was unanimously elected to the chair vacated by the resignation of Dr. Meigs. Dr. Keating brings with him to his new position an enlarged experience as an obstetric practitioner, and great readiness as a lucid and pleasant teacher; having been connected for a number of years with the Philadelphia Medical Association as lecturer on midwifery and the diseases of women and children. There were not less than eleven candidates before the Board of Trustees for this chair.

